# Dr. Hamilton Atchison West

**Published:** 1904-02

**Authors:** 


DR. HAMILTON ATCHISON WEST, of Galveston, Texas, died December 30, 1903, at the residence of his brother, Dr. James N. West, New York city, after a brief illness. Dr. West was one of the most prominent physicians in the southwest, where he acquired a large practice, became secretary of the board of health, and was, for many years, secretary of the Texas State Medical Society. He was a native of Kentucky, where he was born in 1849, and graduated from the medical department of the University of Louisville in 1872. Dr. West made a special study of yellow fever, dengue, and typhoid fever, being often quoted as authority on the former disease. He was formerly professor of the theory and practice of medicine in the medical department of the University of Texas. He served as vice-president of the American Medical Association in 1898. His death is a sad blow to the medical profession in the southwest, as well as to his host of friends throughout the country.

